# The ANDROMEDA prospective cohort study: predictive value of combined criteria to tailor breast cancer screening and new opportunities from circulating markers: study protocol

**DOI:** 10.1186/s12885-017-3784-5

**Published:** 2017-11-22

**Authors:** Livia Giordano, Federica Gallo, Elisabetta Petracci, Giovanna Chiorino, Nereo Segnan, the Andromeda working group, Caterina Anatrone, Franca Artuso, Marcella Beraudi, Denise Casella, Matilde Ceresa, Francesca Di Stefano, Marta Dotti, Simona Feira, Alfonso Frigerio, Francesca Garena, Pamela Giubilato, Maria Piera Mano, Vincenzo Marra, Andrea Menardi, Andrea Ortale, Stefania Pelosin, Andrea Pezzana, Sabina Pitarella, Antonio Ponti, Francesca Saba, Viviana Vergini, Piera Vicari, Salad Heddine Ayoubi, Alessandra Debianchi, Elisabetta Favettini, Ilaria Gregnanin, Lorella Iacazio, Maurizia Mello-Grand, Paola Ostano, Pietro Presti

**Affiliations:** 1 Centre for Cancer Prevention (CPO Piemonte), Unit of Epidemiology and Screening, AOU Città della Salute e della Scienza of Turin, Via Cavour 31, 10123 Turin, Italy; 2 Unity of Biostatistics and Clinical Trials, Istituto Scientifico Romagnolo per lo Studio e Cura dei Tumori, IRCCS, Meldola, Italy; 3 Edo & Elvo Tempia Foundation, Biella, Italy

**Keywords:** Breast cancer, Tailored screening, Risk prediction models

## Abstract

**Background:**

In recent years growing interest has been posed on alternative ways to screen women for breast cancer involving different imaging techniques or adjusting screening interval by breast cancer risk estimates. A new research area is studying circulating microRNAs as molecular biomarkers potentially useful for non invasive early detection together with the analysis of single-nucleotide polymorphisms (SNPs).

The Andromeda study is a prospective cohort study on women attending breast cancer screening in a northern Italian area. The aims of the study are: 1) to define appropriate women risk-based stratifications for personalized screening considering different factors (reproductive, family and biopsy history, breast density, lifestyle habits); 2) to evaluate the diagnostic accuracy of selected circulating microRNAs in a case-control study nested within the above mentioned cohort.

**Methods:**

About 21,000 women aged 46–67 years compliant to screening mammography are expected to be enrolled. At enrolment, information on well-known breast cancer risk factors and life-styles habits are collected through self-admistered questionnaires. Information on breast density and anthropometric measurements (height, weight, body composition, and waist circumference) are recorded. In addition, women are requested to provide a blood sample for serum, plasma and buffy-coat storing for subsequent molecular analyses within the nested case-control study. This investigation will be performed on approximately 233 cases (screen-detected) and 699 matched controls to evaluate SNPs and circulating microRNAs. The whole study will last three years and the cohort will be followed up for ten years to observe the onset of new breast cancer cases.

**Discussion:**

Nowadays women undergo the same screening protocol, independently of their breast density and their individual risk to develop breast cancer. New criteria to better stratify women in risk groups could enable the screening strategies to target high-risk women while reducing interventions in those at low-risk. In this frame the present study will contribute in identifying the feasibility and impact of implementing personalized breast cancer screening.

**Trial registration:**

NCT02618538 (retrospectively registered on 27–11-2015.)

**Electronic supplementary material:**

The online version of this article (10.1186/s12885-017-3784-5) contains supplementary material, which is available to authorized users.

## Background

Breast cancer (BC) represents the most frequent neoplasm in women worldwide, with nearly 1.7 million new cases diagnosed in 2012 [1]*.* In Italy about 1 out of 3 malignant cancers in women (30%) is a BC (with the exception of cutaneous tumors) as reported by Italian cancer registries between 2008 and 2012. They also estimated that in 2016 about 50,000 women would have been diagnosed with BC [2]*.* Mammography is the preferred screening test for early detection of breast cancer and has been studied in more than 600,000 women in 10 randomized trials over the past 50 years [3].

In Italy, mammography screening for early diagnosis has been implemented on a regional basis in several Italian areas. In compliance to national and international screening guidelines, most programs invite women aged 50–69 years to undergo a mammography, every two years [4].

Although mammography has become a standard of detection in BC screening, its limitations are well recognized. In the last decade, mammography BC screening has been the subject of controversy, with several researchers questioning whether the benefit in terms of mortality reduction is large enough to justify the recognized harms of screening, in particular over-diagnosis [5, 6]. Other researchers have provided overviews of the accumulated evidence and concluded that the pros outweigh the cons [7]. The ongoing discussion has led to a lack of clear guidance to both women and their physicians as to whether women should attend mammography screening.

In virtually all population-based BC screening programs, the only risk factor – even though the strongest – used to define the target population is age. This ‘one-size-fits-all’ screening approach has been criticized, as information are gradually becoming available on the advantages of personalized screening, based on appropriate risk stratification [8–14].

A tailored approach can make screening for BC more effective and efficient by targeting women at higher risk, who are most likely to benefit, and reducing exposure to screening in those women at lower risk, who are more likely to experience the harms.

The development of a comprehensive risk prediction model with improved discriminatory power over current models to classify the population into meaningful risk groups will enable the screening strategies to target those at high-risk while reducing interventions in those at low-risk.

The alternative of adding to the existing risk model information on breast density, on life style risk factors (weight and physical activity levels) and on the presence of sensitive and specific minimally invasive biomarkers associated with early neoplastic changes is currently being studied [11–14].

Recently biomedical research has addressed great efforts in evaluating the role of single-nucleotide polymorphisms (SNPs) and microRNAs (miRNAs) in BC risk. The potential use of such molecules for diagnostic/prognostic purposes in regard to BC has been extensively evaluated and with regards to miRNAs, their stability in body fluids has opened new opportunities for anticipating BC diagnosis [15–20] with minimally invasive intervention, especially for women at higher risk [21–23].

Moreover, innovative imaging modalities, such as tomosynthesis [24], are currently under investigation and will yield new knowledge that will need to be incorporated when redesigning screening strategies, especially for women with dense breasts.

In order to assess BC risk over time as accurately as possible, all known and newly identified risk factors for BC need to be assessed. Adding all these information to existing risk models will take us beyond the current state-of-the-art.

Furthermore, once new comprehensive risk prediction methods will have been developed, these risk estimates could be combined with empirical data from primary prevention trials and screening outcomes.

The main aim of the ongoing Andromeda study is to estimate the potential impact of implementing personalized BC screening in order to reduce the still increasing burden of this disease in women attending BC screening.

## Methods/Design

### Study aims

The Andromeda study was designed with two main aims: *1)* to define appropriate women risk-based stratifications for personalized screening considering different criteria such as: a model-based risk estimate of absolute BC risk including reproductive, BC family and biopsy history, breast density, lifestyle habits and *2)* to evaluate whether selected circulating miRNAs, previously associated to BC, are significantly altered in the plasma of cancer patients compared to matched healthy controls; and also if they satisfy pre-specified true and false positive rates, considered minimally acceptable in the screening setting.

In order to define groups of women characterized by different BC risk, the above mentioned criteria will be assessed and compared in terms of positive predictive value (PPV), either when considered alone or in combination. Related to the first main aim, secondary objectives are: to measure risk group-specific screening indicators (i.e., detection rate, recall rate and benign/malignant surgical biopsy ratio); to quantify the impact of tailored screening interventions on health outcomes; to evaluate the economic and organizational feasibility of these interventions.

With regards to the second main aim, secondary objectives are: to assess the tumor characteristics associated with miRNA levels in case subjects to understand the role of these biomarkers in cancer detection; to assess the association between the investigated BC risk factors and miRNA levels in the control group to define different thresholds for screening positivity; to evaluate the presence of 77 established SNPs associated with BC risk [18] and their impact on BC score calculation.

### Study design

The Andromeda study is an ongoing multicentre prospective cohort study on women attending BC screening in two centers in Italy with a nested case control design.

### Study setting

The eligible population of the study consists of 46–67-year-old women invited to breast screening in the cities of Turin and Biella (two Northern Italian cities in Piedmont), where BC screening is a long-standing practice well known by the people living in the area [4]. For organizational reasons the Andromeda study is conducted simultaneously with another investigation, the Proteus study (NCT02590315), a randomized controlled trial aimed at comparing the performance of digital breast tomosynthesis (DBT) with those of standard digital mammography (DM). The study duration is planned for three years. Enrolment started in July 2015 and by the end of the recruitment phase (December 2017) about 21,000 women are expected to be included in the study. The women cohort will be followed up for additional ten years after the study ending through the screening archives to observe the onset of new BC cases.

### Enrolment

**Fig. 1:**
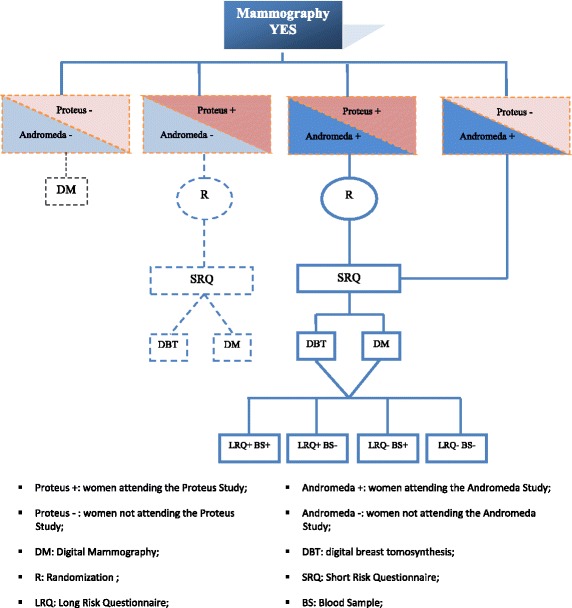
The Andromeda Study

At the time of BC screening appointment, all eligible women are offered to participate in both the Andromeda and the Proteus trials. In Fig. 1 the study flow is reported. After full explanation of the study protocols, a member of the research group obtains written informed consent from each participant (for one or for both studies). Women are reassured that non participation will not impact on their screening path.

Women who agree to participate in Andromeda are asked to fill in a short risk questionnaire (SRQ - Additional file 1) to collect information on general BC risk factors (reproductive and BC family history, previous breast biopsies, basic physical activity level, body mass index and alcohol consumption), immediately at the enrolment desk.

In addition, they are asked to fill in a long risk questionnaire (LRQ - Additional file 2) on diet, physical activity, smoking habits, general state of health and psychological distresses (sections on physical activity and on dietary habits were modified from the EPIC questionnaires [25], the section on psychological distresses was taken from the Brief COPE questionnaire [26]). Women are also invited to undergo anthropometric measurements (height, weight, body composition, and waist circumference) and to provide a blood sample for serum, plasma and buffy-coat storing. Blood specimens are aliquoted, processed and stored at −80 °C. These last procedures can be performed at the time of the examination or at a later date, to be agreed with the woman; fasting is not required.

Mammograms derive either from DM or from DBT (according to the Proteus trial randomization) and are read by two expert radiologists. Breast density is calculated during breast examination, through a specific algorithm [27] and it is classified into categories by means of the Breast Imaging Reporting and Data System (BI-RADS) [28].

### Biomarkers analysis

The Andromeda study includes molecular analyses to evaluate circulating miRNAs and SNPs that will be performed blindly on an appropriate case-control sample extracted from the cohort of enrolled women, as soon as a minimum number of BC cases will be reached. Blinding allows avoiding that knowledge of subject’s outcome status affect the interpretation of an assay result or the care with which the specimen is handled. The prospective specimen collection plus retrospective blinded-evaluation (PRoBE) design assumed here has been proposed by Pepe et al. [29] as a good practice to follow for the pivotal evaluation of biomarker accuracy.

The selected miRNAs come from two consolidated studies [20, 21], and are suited to the screening nature of the Andromeda study.

In more detail the biomarkers to be analyzed are the following:

- Circulating total RNA extraction from plasma samples will be carried out with miRNeasy serum/plasma minikit (Qiagen) with minor modifications of the Exiqon protocol, implying use of a RNA carrier (batteriofage MS2 RNA, Roche Diagnostics®) to promote RNA precipitation. Cel-miR-139 will be used as spike-in control to check extraction yield and to normalize data. RNA samples will be purified on membranes and eluted with nuclease free water and stored at −80 °C. We will carefully evaluate the presence of haemolysis in our analysis and exploit our huge sample cohort to validate previous results on an independent dataset.
- RT-qPCR analysis of selected markers (genes, microRNAs, lncRNAs)
  Total RNA samples will be reverse-transcribed and amplified with specific assays for cel-miR-39, hsa-miR-1228, hsa-miR-145, hsa-miR-451, hsa-miR-222, hsa-miR-18a, hsa-miR-181a and analyzed on a qPCR apparatus with specific dedicated software. Every RNA samples will be analyzed in triplicate. Normalization of expression data will be carried out using hsa-miR-1228 [30] and cel-miR-39 as normalizers. As for SNPs also for miRNAs research findings are continuously updated, so they will be selected considering the most recent findings at the time of their extraction [31].
- SNP genotyping
  Genomic DNA will be extracted from buffy coat with High Pure PCR Template preparation kit (Roche Diagnostics®). 77 SNPs described in Mavaddat et al. [18] will be evaluated using the Personal Genome Machine (Ion Torrent) NGS apparatus.

### Statistical power and analysis

All the data collected through the SRQ will be used to obtain the risk estimates based on a risk prediction model that has been developed for the Italian population and validated on an independent cohort of Italian women (Petracci score) [32].

This information, along with the information on breast density, lifestyle related risk, polygenic risk score from miRNAs and SNPs will be modeled in respect of BC risk factors onset.

As they are determined on the same subjects, among women with abnormal mammography, the most efficient estimate of the ratio between relative (positive vs negative) positive predictive values (PPV) ratios, for any couple of the above factors is given by:

$$ PPV\; ratios=\frac{cancers\;\left(\kern0em positive to factor1\; but\; not to factor2\right)\kern0.5em \times \kern0.5em non cancers\;\left(\kern0em positive to factor2\; but\; not to factor1\right)}{cancers\;\left(\kern0em positive to factor2\; but\; not to factor1\right)\kern0.5em \times \kern0.5em non cancers\;\left(\kern0em positive to factor1\; but\; not to factor2\right)} $$

Assuming that 40% of 21,000 enrolled women have a dense breast, 20% a certain Petracci score, 50% a lifestyle risk and 20% a given miRNA, that 11‰ of study women carry BC and that the overall PPV of mammography is 20%, some 100–120 discordant cancers and 640–740 discordant women with abnormal mammography but no cancer are expected for the considered couples of factors. Under these assumptions, the study has about 80% power to reject (alpha = 0.05) the null hypothesis that the relative PPV ratio equals 1 if its true value is at least 1.8–2.0, depending on the couple of factors considered.

To calculate the sample size necessary for biomarkers pivotal evaluation (expressed on a continuous scale), minimally acceptable and desirable levels of typical performance measures of interests, such as the true-positive rate (TPR) and the false positive rate (FPR) have been defined. For general population screening, the FPR must be quite low to avoid huge numbers of people undergoing unnecessary costly medical procedures. Thus a maximally acceptable false-positive rate (FPR0) of 4% (the minimally acceptable specificity is therefore 96%) and a minimally acceptable sensitivity of 80% (TPR0) were hypothesized. The null hypothesis to be rejected is the following: H0: TPR0 ≤ 0.80 or FPR0 ≥ 0.04.

The sample size required with an 80% power (alpha = 5%) and assuming desirable true-positive rate and false-positive rate of TPR1 = 0.90 and FPR1 = 0.05, respectively, was of 179 cases and 537 controls. Sample size computation was based on theory on the Receiver Operating Characteristic (ROC) curve.

Assuming an annual BC detection rate (DR) of 0.006 in the first year and 0.005 in the second year of the study (therefore the biennial DR considered was 0.011) a total of 233 case patients in the first two years of the study are expected to be observed, thus exceeding the 179 required. These estimates are based on DRs observed in the previous years in the same centers considered in this study (Turin, Biella) [4]. Thus, the molecular analyses will be performed on 233 cases and 699 matched controls. For each case, three control subjects will be selected from the cohort of women, on the basis of the following criteria: no history of cancer, similar age at enrolment (within 5 years), similar race, availability of blood sample, similar date of blood draw.

Moreover, circulating miR-18a, miR-181a, and miR-222 levels - derived from the sister study cohort [21] - on plasma collected from all the women enrolled that have at least one first degree relative with BC will be assessed. The estimated number of this subgroup is 1800 women, 40 of which are expected to develop BC within 18 months, and will therefore belong to the case group.

To assess the predictive ability of the considered criteria (i.e., breast density, absolute risk of developing BC and, lifestyles) in identifying groups of women with different risk levels, the positive and negative predictive values and ratios of predictive values (PPV and PPV ratios) will be calculated for each criterion.

The discrimination ability of single biomarkers will be evaluated by means of the ROC curves, whereas to test if biomarker levels are significantly higher or lower in cases and controls, logistic regression models will be used. To evaluate the association between women’s characteristics or tumor features and biomarker level, linear regression models will be adopted.

## Discussion

This study has been designed to achieve several outcomes: to collect necessary information to compare performance of BC evaluation criteria; to acquire necessary data to design stratified BC screening programs; to evaluate feasibility and impact of different screening strategies; to establish a conspicuous bio-bank useful to accurately validate selected biomarkers (and future novel ones) .

Although follow up is planned for 10 years, preliminary results will be available at the end of the three years of study duration. The above mentioned results could contribute in defining BC screening protocols on different risk factors other than age only, as it currently is.

Securely a critical aspect of the study could be represented by the acceptance of women to undergo a blood test. This point, though difficult, is a key point of the project as it will allow us to establish the bio-bank. To try and overcome these barriers and encourage women participation we are intensely working on communication and organization.

As concerns communication, very detailed information materials strongly emphasizing the goal of blood samples collection and its impact on the final outcomes of the study have been arranged. This material clearly explains the relevance of the personal history of each woman in terms of potential BC risk factors, so encouraging participants in following the whole study path.

The information material is designed to encourage, in a friendly and direct way, women to provide information about their lifestyle and a blood sample, so contributing to improve BC prevention strategies: a precious gift not only to themselves but also to other women.

Furthermore, a call centre has been be set up allowing women to get further information and to speak directly with a dedicated health operator. Particular attention is given to the training of health personnel interacting with women at the time of the screening, making them able to provide adequate and correct information. In particular, since the screening invitation letter is signed by GPs, they receive special educational interventions.

Regarding logistic aspects, we are giving participants the opportunity either to undergo blood drawing immediately after mammography or to fix a suitable appointment, facilitated by the fact that fasting is not necessary.

Particular attention is given to the formulation and administration of the LRQ, taking care to avoid overlap with the SRQ, reducing compilation times and trying to get standardized and comparable answers. The SRQ has been specifically designed for tablet devices to minimize both time and errors in inputting data.

Another concern is related to the molecular analyses. Since we do not know if specific miRNA levels fluctuate according to age or other demographical parameters or smoking exposure, we plan to use age and smoking matched patients without any diagnosis of (pre)malignant disease as controls for the miRNA analysis on plasma samples.

To face problems related to evaluation of miRNA expression particular caution is adopted especially regarding sample handling, timing and procedure of plasma separation, storage prior to extraction and protocols for RNA extraction and yield control. Our measures should be reliably translated in a screening context, therefore we analyze microRNAs already tested in prospective studies on plasma of cases before diagnosis and on matched controls, and validated using NGS approaches or Taqman on gene cards on independent cohorts.

A strength point of the study is represented by the prospective uniform nature of specimen collection for all subjects from a single cohort. This eliminates systematic biases by ensuring that case patients and control subjects are collected in exactly the same way and, in this case, from the setting for which biomarker is intended, that is the standard screening setting. Moreover, collecting and storing specimens before ascertaining the clinical diagnosis may provide information on the time dimension, that is, if the levels of the biomarker deviate from those in control subjects close to the time of clinical diagnosis, then the biomarker shows little promise for screening. On the other hand, if levels in case subjects reach levels distinct from those in controls years before clinical diagnosis, then the biomarker’s potential for early detection is increased.

Finally, the retrospective component of the design also avoids ethical problems, such as knowing the biomarker value without knowing how it should affect patient care. Moreover, sample storing will allow us future discovery, if more standardized and reliable techniques will become available, of novel circulating molecules whenever those selected will not satisfy our expectance.

## Additional files

Additional file 1: Short Risk Questionnaire - SRQ. (PDF 29 kb)

Additional file 2: Long Risk Questionnaire - LRQ. (PDF 6689 kb)

## Abbreviations

BC: Breast cancer
BS: Blood sample
DBT: digital breast tomosynthesis
DM: Digital mammography
DR: Detection rate
FPR: False positive rate
LRQ: Long risk questionnaire
MiRNA: microRNA
PCR: Polymerase chain reaction
PPV: Positive predictive value
PRoBE: Prospective specimen collection plus retrospective blinded-evaluation
R: Randomization
ROC: Receiver operating characteristic curve
SNP: Single-nucleotide polymorphism
SRQ: Short risk questionnaire
TPR: True positive rate
