# Supplementary material for: The ANDROMEDA prospective cohort study: predictive value of combined criteria to tailor breast cancer screening and new opportunities from circulating markers: study protocol
Source: BMC Cancer. 2017 Nov 22;17:785. doi: 10.1186/s12885-017-3784-5 (PMC5700540; doi:10.1186/s12885-017-3784-5)
Supplement: Supplementary file 1 — Short Risk Questionnaire - SRQ. (PDF 29 kb) [file 12885_2017_3784_MOESM1_ESM.pdf]

Studio ANDROMEDA

SHORT RISK QUESTIONNAIRE

(to be filled on the tablet device)

1) GENERAL INFORMATION

1.1 Identification number

____

1.2 Please insert the following general information:

Height in cm ___________ Weight in kg ____________

1.3 What is your study level?

(Select one option only)

- I have never studied
- Primary school license
- Secondary school license
- Professional school diploma (2/3 years course)
- High school diploma (4/5 years course)
- Degree (3 years course)
- Master degree (4/5 years course)
- PhD

2) PERSONAL HISTORY

2.1 How many among mother, daughters, and sisters had a breast cancer diagnosis?

- None
- One
- Two or more
- I don’t know / I don’t remember

2.2 Have you ever had a breast biopsy (including needle biopsy) in the past?

- None
- One
- Two or more
- I don’t know / I don’t remember

2.3 *(If yes)* How many biopsies have you undergone to?

- 1
- 2
- 3
- 4
- more than 4

3) HORMONAL AND REPRODUCTIVE HISTORY

3.1 At what age your menstrual cycle started?

- 11 years or less
- 12/13 years
- 14 years or more
- I don’t know / I don’t remember

3.2 Do you still have your menstrual cycle?

- Yes
- No

3.3 *(If you still have your menstrual cycle)* How many menstrual cycles have you had in the past 12 months?

- 1-3
- 4-5
- 6-9
- 10 or more
- I don’t know / I don’t remember

3.4 *(If you do not have your menstrual cycle any more)* At what age did you have your last menstrual cycle?
________________________ *(Please, leave blank if you don’t know or can’t remember)*

3.5 How many children did you have? (born alive)

- I have no children
- 1
- 2
- 3
- more than 3

3.6 *(If you had children)* At what age did you have your first child? (born alive)

- 19 years or less
- 20-24 years
- 25-29 years
- 30 years or more

3.7 Are you currently under hormonal therapy?

- Yes
- No

4) HABITS

4.1 When you were 30-39 years, which was your level of physical activity at work or at home (if housewife)?

- very heavy *(e.g., construction worker, athlete or dancer)*
- heavy *(e.g., farmer, workwoman)*
- medium *(e.g., waitress, nursery school teacher)*
- standing up *(e.g., shop assistant, teacher)*
- mainly sitting *(e.g., office worker, student)*

4.2 When you were 30-39 years, how many hours a week of physical activity did you practice in your free time?

- more than 7 hours/week
- 5-7 hours/week
- 2-4 hours/week
- less than 2 hours/week

4.3 Do you drink alcoholic drinks or did you drink them in the past?

- Yes *(also occasionally)*
- No *(I never drank them)*
- In the past *(now, I don't drink them)*

4.4 *(If you drank alcoholic drinks in the past but not anymore)* How long ago have you given up?

- less than 1 year
- more than 1 year
