# Supplementary material for: The ANDROMEDA prospective cohort study: predictive value of combined criteria to tailor breast cancer screening and new opportunities from circulating markers: study protocol
Source: BMC Cancer. 2017 Nov 22;17:785. doi: 10.1186/s12885-017-3784-5 (PMC5700540; doi:10.1186/s12885-017-3784-5)
Supplement: Supplementary file 2 — Long Risk Questionnaire - LRQ. (PDF 6689 kb) [file 12885_2017_3784_MOESM2_ESM.pdf]

Patient identification code

Center: ____

Studio
ANDROMEDA

QUESTIONNAIRE ON LIFE STYLE HABITS

This questionnaire can be:

- **filled in and delivered to the project staff at the screening center;**
- **filled in at home and sent to San Giovanni Hospital, Via Cavour 31, 10123 Turin, or delivered to the project staff at ground floor;**
- **filled in at home and returned at the time of the appointment for the blood sample**

If you have any questions about the study you can telephone on the free toll number 800.00.51.41

Surname ________________________

Forename (s) ____________________________

Place of birth __________________________________ **Province:**_________________

Date of birth _______ / _______ / _________

Mother place of birth_________________________ **Province:**_________________

Father place of birth_________________________ **Province:**_________________

Marital status (please tackle the right answer)

	conjugated or cohabiting
	unmarried
	widow
	separated or divorced

Date of compilation _______ / _______ / _________

Section 1 – PHYSICAL ACTIVITY

The questions of this questionnaire refer to activities carried out <u>during the last year</u>. *Please answer to all the questions.*

JOB

1-1) Do you have a paid job at present?

☐ YES, full time ☐ YES, part time ☐ NO

If YES, what is your job? ____

If no, NO you are currently:

☐ **Retired** **what job did you do before? __________________**

☐ **Housewife**

☐ **Not occupied** **what job did you do before? ______________**

1-2) In this job or past job, which of the following best describe your physical activity? Please tick one only.

☐ **SEDENTARY OCCUPATION** – You spent most of your time sitting (such as in an office)

☐ **STANDING OCCUPATION** – You spent most of your time standing or walking. However, your work does not require intense physical effort (e.g. shop assistant, hairdresser, guard).

☐ **MANUAL WORK** – This involves some physical effort including handling of heavy objects and use of tools (e.g. plumber, electrician, carpenter)

☐ **HEAVY MANUAL WORK** – This involves very vigorous physical activity including handling very heavy objects (e.g. docker, miner, bricklayer, construction worker)

1-3) Generally, how many hours a day do you spend for housekeeping?

☐ Less than 1 hour ☐ 1-2 hours ☐ 3–4 hours ☐ 5-6hours ☐ 7 hour or more

1-4) How many stairwells do you climb every day on average? Consider it at home and at work. (For example: Work or work on the 4th floor and I go twice = 8 levels of stairs)

☐ none ☐ 1 – 2 ☐ 3 – 4 ☐ 5 – 6 ☐ 7 - 9

☐ 10 - 12 ☐ 13 – 15 ☐ 16 – 20 ☐ 21 floors or more per day

PHYSICAL ACTIVITY DURING YOUR FREE TIME (in any case not at work)

In a typical week, during the last year, how many hours did you spend on each of the following activities?
Please, answer to all the questions, even if the answer is 'never'..

1-5) Walking (going to work, doing shopping, taking a walk)

IN SUMMER

☐ Never ☐ Less then 1 hour per week ☐ 1-2 hours per week ☐3-4 hours per week

☐ 5-6 hours per week ☐ 7-8 hours per week ☐ 9-10 hours per week ☐ 11 hours or more per week

IN WINTER

☐ Never ☐ Less then 1 hour per week ☐ 1-2 hours per week ☐3-4 hours per week

☐ 5-6 hours per week ☐ 7-8 hours per week ☐ 9-10 hours per week ☐ 11 hours or more per week

1-6) Cycling (going to work, in your free time)

IN SUMMER

☐ Never ☐ Less then 1 hour per week ☐ 1-2 hours per week ☐3-4 hours per week

☐ 5-6 hours per week ☐ 7-8 hours per week ☐ 9-10 hours per week ☐ 11 hours or more per week

IN WINTER

☐ Never ☐ Less then 1 hour per week ☐ 1-2 hours per week ☐3-4 hours per week

☐ 5-6 hours per week ☐ 7-8 hours per week ☐ 9-10 hours per week ☐ 11 hours or more per week

1-7) Gardening / Do–it-yourself activities

IN SUMMER

☐ Never ☐ Less then 1 hour per week ☐ 1-2 hours per week ☐3-4 hours per week

☐ 5-6 hours per week ☐ 7-8 hours per week ☐ 9-10 hours per week ☐ 11 hours or more per week

IN WINTER

☐ Never ☐ Less then 1 hour per week ☐ 1-2 hours per week ☐3-4 hours per week

☐ 5-6 hours per week ☐ 7-8 hours per week ☐ 9-10 hours per week ☐ 11 hours or more per week

1-8) Sport (including gym, dance, swimming, tennis, racing, hiking, cycling, mountain biking)

IN SUMMER

☐ Never ☐ Less then 1 hour per week ☐ 1-2 hours per week ☐3-4 hours per week

☐ 5-6 hours per week ☐ 7-8 hours per week ☐ 9-10 hours per week ☐ 11 hours or more per week

IN WINTER

☐ Never ☐ Less then 1 hour per week ☐ 1-2 hours per week ☐ 3-4 hours per week

☐ 5-6 hours per week ☐ 7-8 hours per week ☐ 9-10 hours per week ☐ 11 hours or more per week

1-9) Do you practice any activities vigorous enough to cause sweating or a faster heartbeat?

Yes☐ NO ☐

If yes, for how many hours each week?

☐ Never ☐ Less then 1 hour per week ☐ 1-2 hours per week ☐3-4 hours per week

☐ 5-6 hours per week ☐ 7-8 hours per week ☐ 9-10 hours per week ☐ 11 hours or more per week

1-10) In the last year, did you change the amount of time you spend on physical activity significantly?

Yes ☐ NO ☐

If yes, you have:

☐ reduced your time ☐ increased your time

If you have reduced or increased the time spend on physical activity, please specify the reason:

☐ health reasons (please specify)____

☐ other reasons (please specify)____

Section 2 – DIETARY HABITS

The following questions refer to your eating habits in the last year.

In answering the questions, please think about all meals you eat in a day (breakfast, lunch, dinner and snacks)
Remember: answer to ALL the questions, even if the answer is NEVER, as in the following example:

2-8) How many times did you eat red beef (including calf), sheep, pig, horse?

Times per day____	Times per week 3	Times per month___	Times per year___	☐ Never

Usually your dish was:

☐ smaller ☒ like this ☐ like this ☐ like this ☐ larger

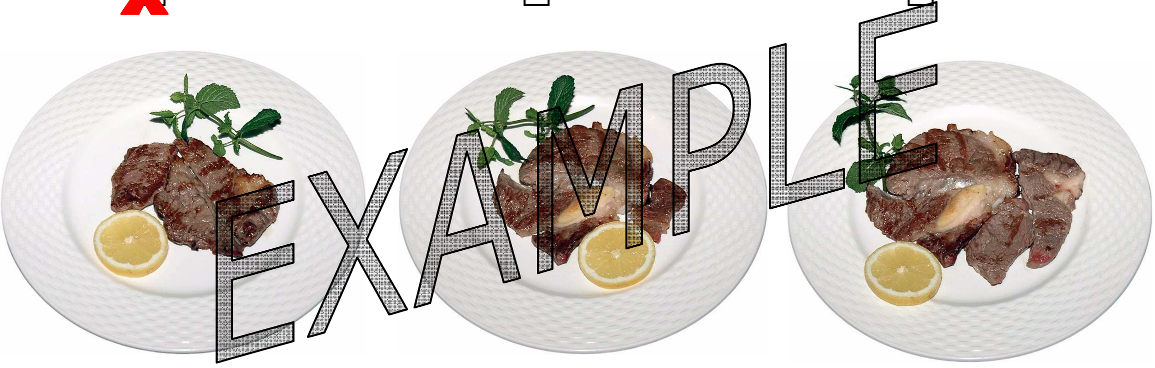

How was usually prepared the meat you ate?

	Very often	Often	Sometimes	Rarely	Never
grilled, roasted, boiled, raw	X				
stewed	X				
stuffed meat, meatballs, fried					X
with oil, with butter, scallops				X	

The example shows that the consumption of red meat is three times a week and that the portion usually consumed is the same as the one reproduced in the first of the three photos.

Please indicate the portion you eat in all answers. The only case that should not be mentioned is when the answer is NEVER.

Concerning the preparation type, the example shows that it is eaten "very often" on the grill grid ... and stewed .... "never" fried ... "rarely" cooked with butter.

FIRST COURSES

2-1) How many times did you eat pasta?

Times per day_____	Times per week__	Times per month___	Times per year___	☐ Never

Usually your dish was:

☐ smaller ☐ like this ☐ like this ☐ like this ☐ larger

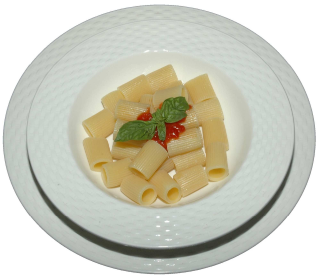

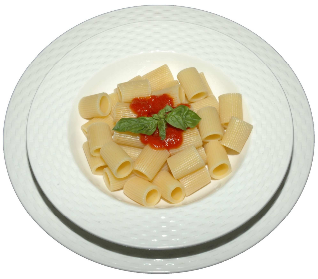

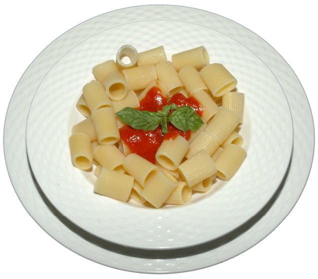

How often did you eat these kinds of pasta?

	Very often	Often	Sometimes	Rarely	Never
durum wheat pasta					
whole wheat pasta					
pasta made from other cereals (kamut, spelt)					
egg pasta (noodles)					
stuffed pasta					

How was usually prepared the pasta you ate?

	Very often	Often	Sometimes	Rarely	Never
with oil or butter, only					
with tomato sauce					
with meat sauce (e.g. with sauerkraut, sausage)					
with vegetables (e.g. broccoli, zucchini, eggplant, pesto sauce)					
fish sauce (e.g. with tuna, salmon, seafood)					
pasta salad					
lasagna, cannelloni					

2-2) How many times did you eat common white rice?

Times per day____	Times per week__	Times per month___	Times per year___	☐ Never

Usually your dish was:

☐ smaller ☐ like this ☐ like this ☐ like this ☐ larger

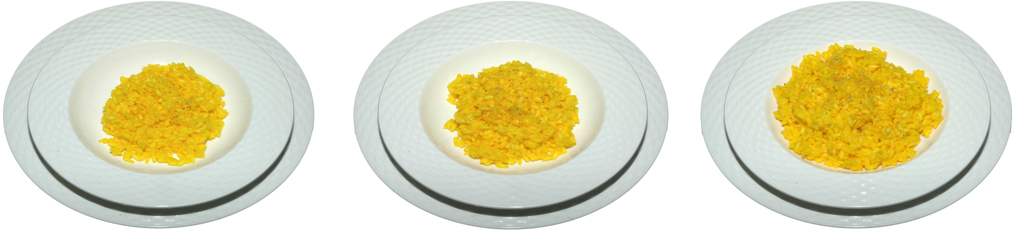

How was usually prepared the rice you ate?

	Very often	Often	Sometimes	Rarely	Never
with oil or butter, only					
with tomato sauce					
with meat sauce (e.g. with sauerkraut, sausage)					
with vegetables (e.g. broccoli, zucchini, eggplant, pesto sauce)					
fish sauce (e.g. with tuna, salmon, seafood)					
rice salad					

2-3) How many times did you eat whole grains (like wheat, spelt, barley, complete rice, buckwheat)?

Times per day____	Times per week__	Times per month___	Times per year___	☐ Never

Usually your plate was:

☐ smaller ☐ like this ☐ like this ☐ like this ☐ larger

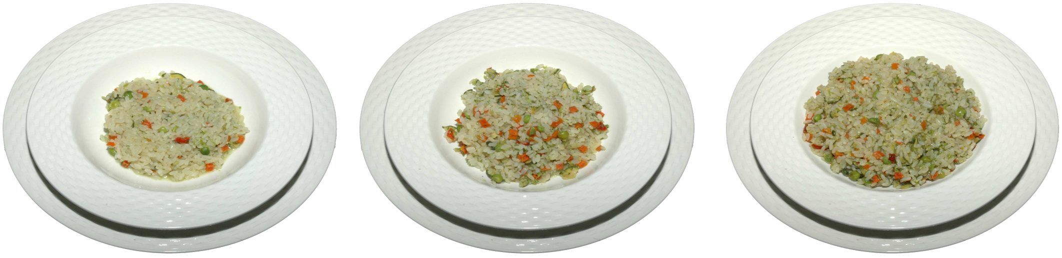

How were usually prepared the cereals you ate?

	Very often	Often	Sometimes	Rarely	Never
with oil or butter, only					
with tomato sauce					
with meat sauce (e.g. with sauerkraut, sausage)					
with vegetables (e.g. broccoli, zucchini, eggplant, pesto sauce)					
fish sauce (e.g. with tuna, salmon, seafood)					
cereal salad					

2-4) How many times did you eat pizza?

Please, consider pizza eaten as a meal, both at home or in a pizza restaurant.

Times per day_____	Times per week__	Times per month___	Times per year___	☐ Never

2-5) How many times did you eat cornmeal mush? Please, consider cornmeal mush eaten as a meal.

Times per day_____	Times per week__	Times per month___	Times per year___	☐ Never

Usually your dish was:

☐ smaller ☐ like this ☐ like this ☐ like this ☐ larger

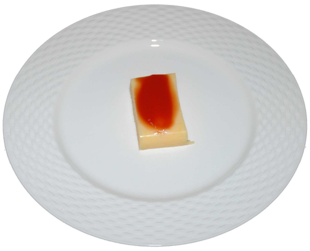
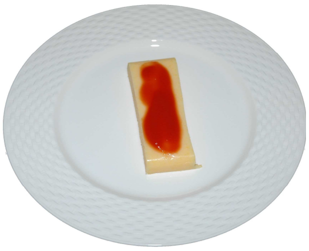
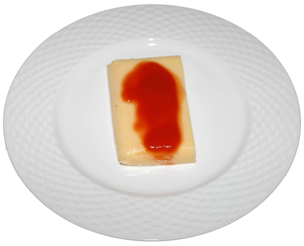

2-6) How many times did you eat vegetable soup/legume soup? With or without some pasta or rice.

Times per day_____	Times per week__	Times per month___	Times per year___	☐ Never

Usually your dish was:

☐ smaller ☐ like this ☐ like this ☐ like this ☐ larger

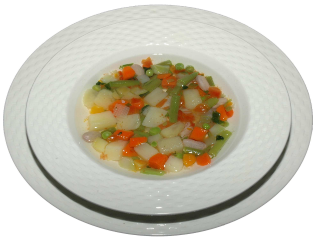
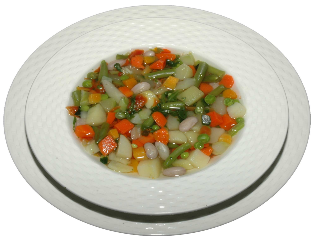
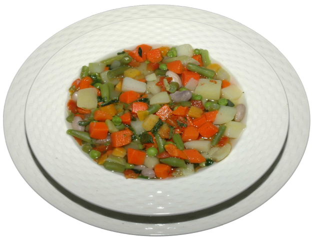

How were usually prepared the soups you ate?

	Very often	Often	Sometimes	Rarely	Never
minestrone, creamy soups					
with beans, chick peas, lentils, broad beans					
consommé					
with pasta, rice or cereals					

MEAT

2-7) How many times did you eat red beef (including calf), sheep, pig, horse?

Times per day_____	Times per week__	Times per month___	Times per year___	☐ Never

Usually, your dish was:

☐ smaller ☐ like this ☐ like this ☐ like this ☐ larger

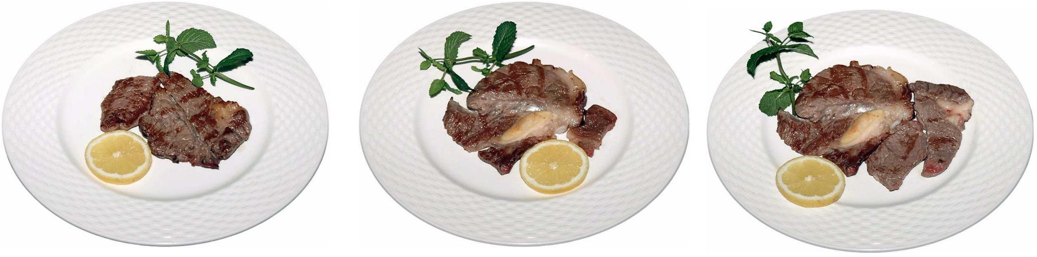

How was usually prepared the red meat you ate?

	Very often	Often	Sometimes	Rarely	Never
grilled, roasted, boiled, raw					
stewed					
stuffed meat, meatballs, fried					
with oil, with butter, scallops					
preserved meat (in jars)					

2-8) How many times did you eat white meat, like chicken, rabbit, turkey?

Times per day_____	Times per week__	Times per month___	Times per year___	☐ Never

Usually your dish was:

☐ smaller ☐ like this ☐ like this ☐ like this ☐ larger

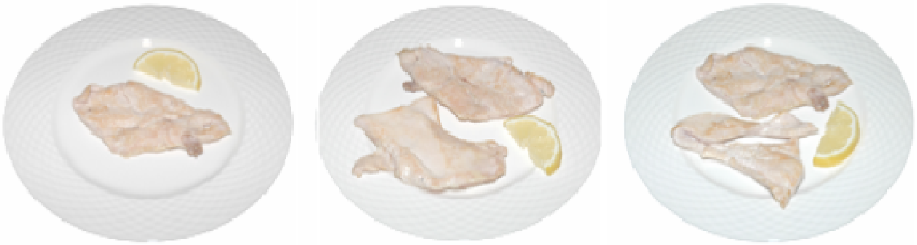

How was usually prepared the white meat you ate?

	Very often	Often	Sometimes	Rarely	Never
grilled, roasted, boiled, raw					
stewed					
stuffed meat, meatballs, fried					
with oil, with butter, scallops					
preserved meat (in jars)					

FISH

2-9) How many times did you eat fish (e.g. trout, hake, sole, salmon, anchovies, mackerel)? Consider both fresh and frozen fish.

Times per day_____ | Times per week__ | Times per month___ | Times per year___ | ☐ Never

Usually your dish was:

☐ smaller ☐ like this ☐ like this ☐ like this ☐ larger

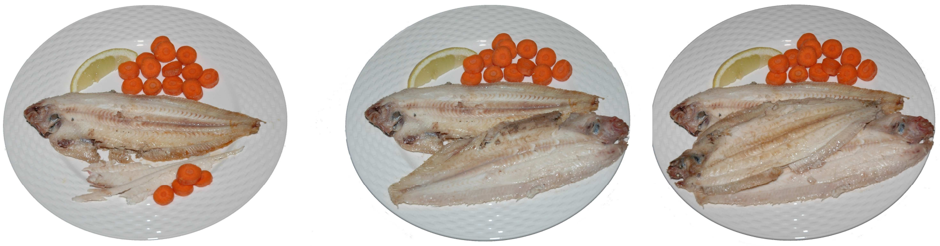

How often did you eat fish?

	Very often	Often	Sometimes	Rarely	Never
tuna, swordfish, shaggy, salmon, cod, hake					
sole, sea bream, sea bass					
mackerel, anchovies, palamites					
trout, pike					

How was usually prepared the fish you ate?

	Very often	Often	Sometimes	Rarely	Never
baked, grilled, irrigated, boiled, raw					
stewed					
fried, fishballs					
with oil, with butter					
canned fish or in jars (in oil or natural)					

2-10) How many times did you eat seafood (e.g., shrimps, scampi, octopus, squid, mussels, clams)? Consider both fresh and frozen ones.

Times per day_____	Times per week__	Times per month___	Times per year___	☐ Never

Usually your dish was:

☐ smaller ☐ like this ☐ like this ☐ like this ☐ larger

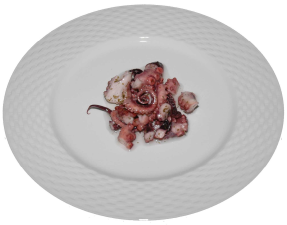
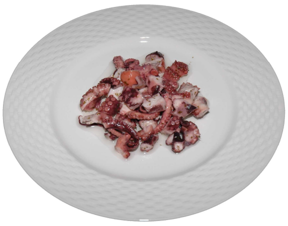
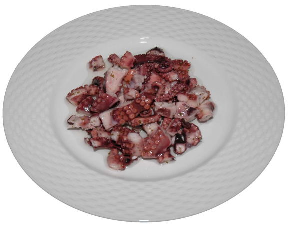

How was usually prepared the seafood you ate?

	Very often	Often	Sometimes	Rarely	Never
baked, grilled, irrigated, boiled, raw					
stewed					
fried, fishballs					
with oil, with butter					
canned fish or in jars (in oil or natural)					

COLD CUTS

In answering the questions, please also consider cold cuts contained in stuffed sandwiches, piadinas, and toasts.

2-11) How many times did you eat cold cuts, like parma ham, cooked ham, bresaola, speck?

Times per day____	Times per week__	Times per month___	Times per year___	☐ Never

Usually your dish was:

☐ smaller ☐ like this ☐ like this ☐ like this ☐ larger

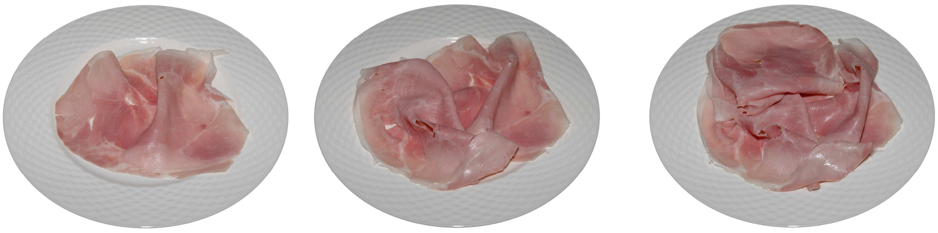

2-12) How many times did you eat cold cuts, like salami, bologna, bacon, sausages, cotechino, würstel?

Times per day____	Times per week__	Times per month___	Times per year___	☐ Never

Usually your dish was:

☐ smaller ☐ like this ☐ like this ☐ like this ☐ larger

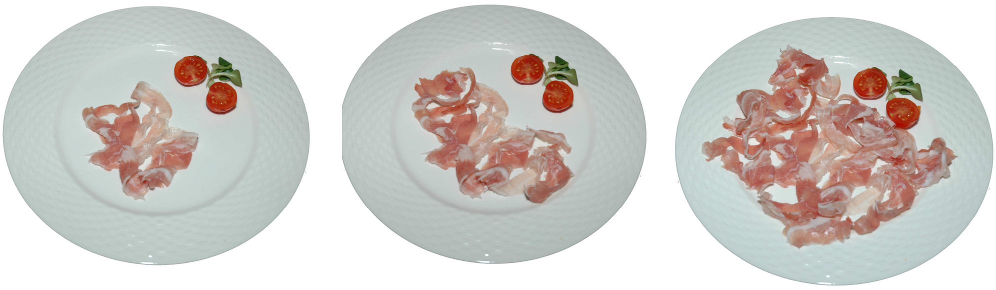

CHEESE

In answering the questions, please also consider cheese contained in stuffed sandwiches, piadinas, and toasts.

2-13) How many times did you eat matured cheese, like fontina, toma, parmesan, provolone, emmenthal, pecorino, caciocavallo, gorgonzola?

Times per day_____	Times per week__	Times per month___	Times per year___	☐ Never

Usually your dish was:

☐ smaller ☐ like this ☐ like this ☐ like this ☐ larger

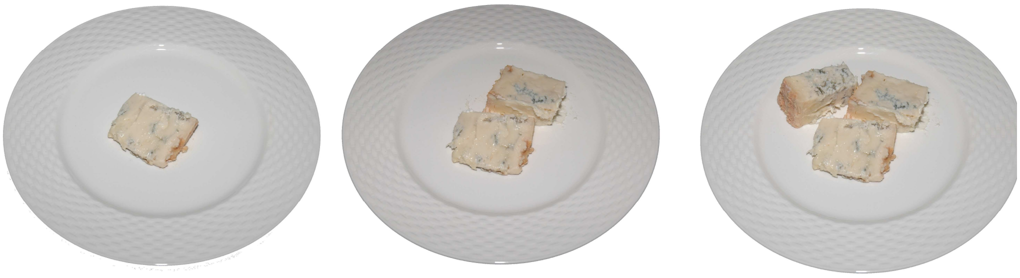

2-14) How many times did you eat fresh/spreadable cheese, like cottage cheese, mozzarella, stracchino, robiola?

Times per day_____	Times per week__	Times per month___	Times per year___	☐ Never

Usually your dish was:

☐ smaller ☐ like this ☐ like this ☐ like this ☐ larger

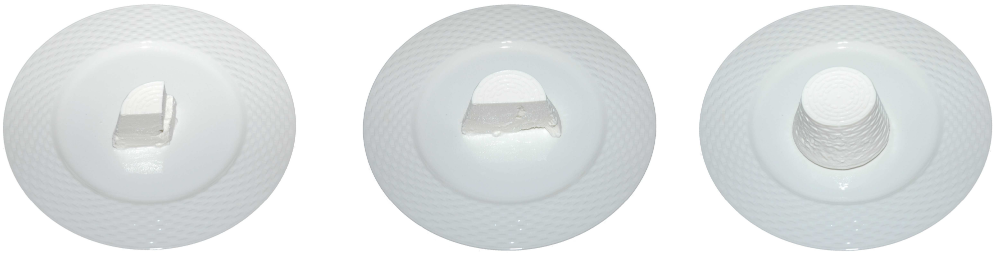

EGGS

2-15) How many eggs did you eat (boiled, fried, omelets)?

Times per day_____	Times per week__	Times per month___	Times per year___	☐ Never

BEANS AND VEGETABLE SUBSTANCES OF ANIMAL PROTEINS

2-16) How many times did you eat legumes, such as beans, lentils, chickpeas, peas, soya beans (fresh, dried, frozen or canned)? In *answering the question, please do not consider legumes eaten in first courses and soups.*

Times per day_____	Times per week__	Times per month___	Times per year___	☐ Never

Usually your dish was:

☐ smaller ☐ like this ☐ like this ☐ like this ☐ larger

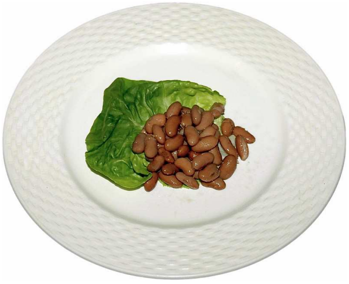

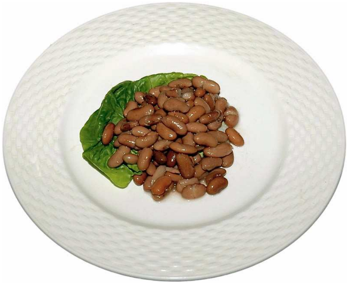

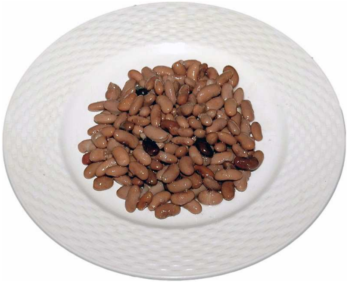

How were usually prepared the beans you ate?

	Very often	Often	Sometimes	Rarely	Never
in cans or in jars					
boiled or steamed					
stewed					
bean balls					
in puree					

2-17) How many times did you eat foods like tofu, miso, tempeh, seitan?

Times per day_____	Times per week__	Times per month___	Times per year___	☐ Never

VEGETABLES

2-18) How many times did you eat cooked vegetables (e.g., cabbage, cauliflower, zucchini, carrots, eggplants, spinaches, leeks, beets)?

Times per day_____ | Times per week__ | Times per month___ | Times per year___ | ☐ Never

Usually your dish was:

☐ smaller ☐ like this ☐ like this ☐ like this ☐ larger

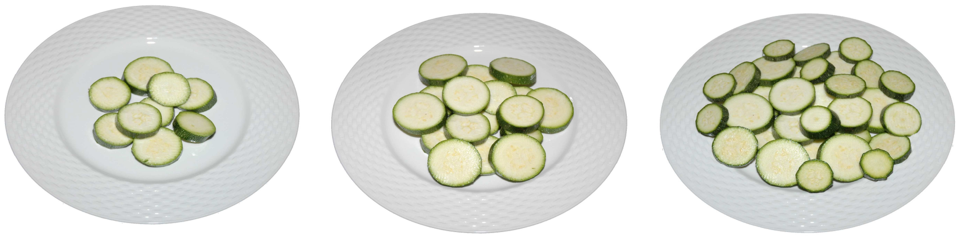

How were usually prepared the vegetables you ate?

	Very often	Often	Sometimes	Rarely	Never
steamed or boiled					
stewed					
dumplings					
grilled or baked					
fried					

2-19) How many times did you eat raw vegetables (e.g., carrots, tomatoes, artichokes, fennel, cabbage)?

Times per day_____ | Times per week__ | Times per month___ | Times per year___ | ☐ Never

Usually, your dish was:

☐ smaller ☐ like this ☐ like this ☐ like this ☐ larger

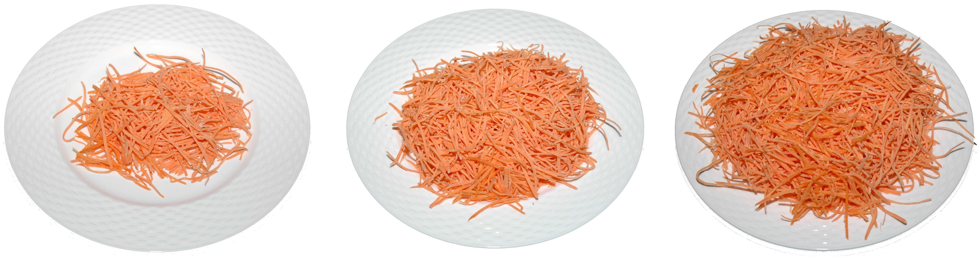

2-20) How many times did you eat raw leafy vegetables (e.g., lettuce, valerian, radicchio, endive)?

Times per day_____ | Times per week__ | Times per month___ | Times per year___ | ☐ Never

Usually your dish was:

☐ smaller ☐ like this ☐ like this ☐ like this ☐ larger

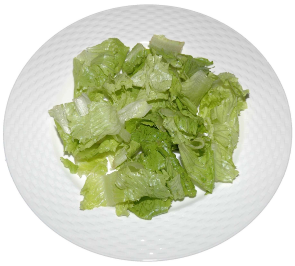
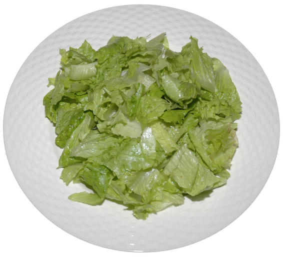
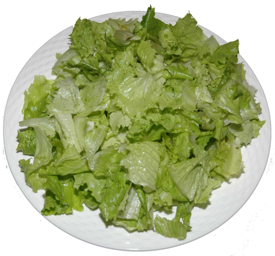

2-21) How many times did you eat potatoes?

Times per day_____ | Times per week__ | Times per month___ | Times per year___ | ☐ Never

Usually your dish was:

☐ smaller ☐ like this ☐ like this ☐ like this ☐ larger

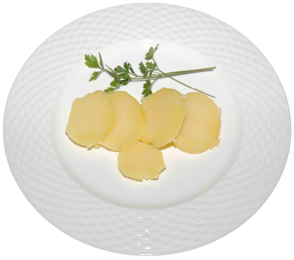
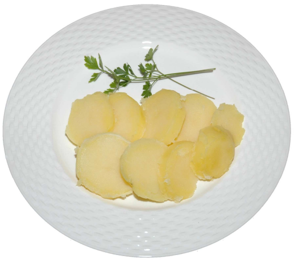
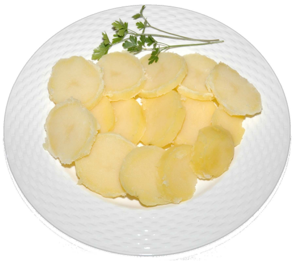

How were usually prepared the potatoes you ate?

	Very often	Often	Sometimes	Rarely	Never
boiled					
stewed					
baked					
mashed					
French fries, chips, potato croquettes					

FRUITS

2-22) How many fresh fruits portions did you eat? (a portion corresponds to an apple or a pear or a cup of strawberries or three tangerines or two kiwis or three apricots)

Times per day_____	Times per week__	Times per month___	Times per year___	☐ Never

Which fruits did you eat?

	Very often	Often	Sometimes	Rarely	Never
apples, pears					
apricots, peaches					
oranges (also orange juice), tangerines, kiwis					
grapes, figs, bananas					
melon, watermelon					
raspberries, blueberries, strawberries					
cherries, plums					
others (specify)					

2-23) How often did you eat fruit salads or dried fruit (e.g., apricots, plums)?

Times per day_____	Times per week__	Times per month___	Times per year___	☐ Never

2-24) How many times did you eat nuts (e.g., walnuts, hazelnuts, peanuts, almonds)? Please, also consider nuts used in cakes.

Times per day_____	Times per week__	Times per month___	Times per year___	☐ Never

Usually your portion was:

☐ smaller ☐ like this ☐ like this ☐ like this ☐ larger

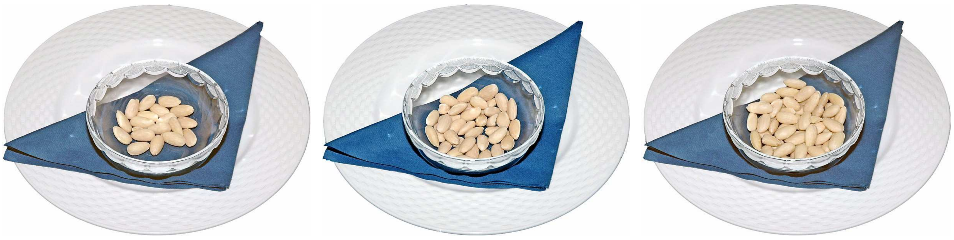

2-25) How many times did you eat seeds (e.g., flax, pumpkin, sunflower, sesame, etc.)? Please, also consider seeds added to bread, salads or consumed as snacks.

Times per day_____	Times per week__	Times per month___	Times per year___	☐ Never

Usually, how many spoonfuls of seeds did you eat?

☐ less than 1 ☐ 1 or 2 ☐ 3 or 4 ☐ more than 4

SEASONINGS

2-26) What kind of fats did you use for:
(Mark an X below the seasoning used most frequently)

	extra virgin olive oil	olive oil	seed oil (e.g., peanuts, sunflower)	margarine and vegetable cream	cream	butter
preparing sauces						
cooking meat or fish						
frying						
flavoring raw vegetables						
flavoring cooked vegetables						

2-27) How often did you use spices (e.g., turmeric, pepper, chili pepper, curry)?

very often ☐ o often ☐ o sometimes ☐ o rarely ☐ o never ☐

2-28) When you ate outside, did you usually add salt to the various dishes?

very often ☐ o often ☐ o sometimes ☐ o rarely ☐ o never ☐

BREAD, CRACKERS, BREADSTICKS

In answering the questions, please think about breakfast and other meals, and outside meals.

2-29) How many portions of bread or similar (crackers, breadsticks, biscuits) prepared with white flour did you eat? (a portion is equal to a slice of white bread or a small sandwich or a cracker package or 3-4 breadsticks).

portions per day ______	o	portions per week ______	o	portions per month ______	o	portions per year ______	o	never ☐

2-30) How many portions of bread or similar (crackers, breadsticks, biscuits) prepared with COMPLETE flour or whole grains did you eat? (a portion is equivalent to a slice of whole wheat bread or a small sandwich or a cracker package or 3-4 breadsticks).

portions per day ______	o	portions per week ______	o	portions per month ______	o	portions per year ______	o	never ☐

2-31) How many portions of bread with added seasonings (such as pizza, focaccia, bread with oil) did you eat?

portions per day ______	o	portions per week ______	o	portions per month ______	o	portions per year ______	o	never ☐

2-32) How many times did you eat savory snacks (e.g., croutons, crisps)?

very often ☐	o	often ☐	o	sometimes ☐	o	rarely ☐	o	never ☐

WINE, BEER, ALCOHOL, DRINKS

2-33) How many glasses of wine did you drink?

N per day ____ o N per week __ o N per month_ o N per year__ o none ☐

Which wine did you drink?

	Very often	Often	Sometimes	Rarely	Never
white wine					
red wine					
rosé wine					
sparkling wine					

2-34) How many cans or bottles (33 cl) of beer did you drink?

N per day ____ o N per week __ o N per month_ o N per year__ o none ☐

2-35) How many shots of spirits, bitter, liqueur (e.g., whiskey, vodka, brandy, rum, grappa) did you drink?

N per day ____ o N per week __ o N per month_ o N per year__ o none ☐

2-36) How many glasses or cans of soft drinks (e.g., fruit juices, orange soda, coke) did you drink?

N per day ____ o N per week __ o N per month_ o N per year__ o none ☐

COFFEE, MILK, YOGURT, HERBAL TEA

In answering the questions, please think about breakfast and other meals, and outside meals.

2-37) How many cups of coffee (even decaffeinated) did you drink?

N per day ___	o	N per week __	o	N per month_	o	N per year__	o	none ☐

2-38) How many cups of cappuccino, coffee with milk, stained milk did you drink?

N per day ___	o	N per week __	o	N per month_	o	N per year__	o	none ☐

2-39) How many glasses of milk or natural yogurt (whole fat, half fat or skimmed) did you drink? (one cup matches two glasses)

N per day ___	o	N per week __	o	N per month_	o	N per year__	o	none ☐

2-40) How many glasses of sweetened milk or sweetened/fruit yogurt (whole fat, half fat or skimmed) did you drink? (one cup matches two glasses)

N per day ___	o	N per week __	o	N per month_	o	N per year__	o	none ☐

2-41) How many glasses of milk and/or vegetable yogurt (e.g., soya, oats, rice) did you drink? (one cup matches two glasses)

N per day ___	o	N per week __	o	N per month_	o	N per year__	o	none ☐

2-42) How many cups of tea, herbal tea or barley coffee did you drink?

N per day ___	o	N per week __	o	N per month_	o	N per year__	o	none ☐

Mostly what kind of tea or similar did you drink?

	Very often	often	sometimes	rarely	never
black or common tea					
green tea					
red tea					
detained tea					
herbal tea					
barley coffee					

2-43) Normally, how many sugars (white or brown) or honeycombs did you put in the following foods? (1 sachet = 2 teaspoons) (if you do not use sugar, please indicate 0)

	N
glass of milk	
glass of vegetable milk	
jar of yogurt	

	N
cup of coffee	
cup of tea, herbal tea or barley coffee	
cup of cappuccino, coffee with milk	

SWEETS

In answering the questions, please think about breakfast and other meals, and outside meals.

2-44) How many breakfast biscuits did you eat?

N per day ___ o N per week __ o N per month_ o N per year__ o none ☐

2-45) How many brioches or croissants did you eat?

N per day ___ o N per week __ o N per month_ o N per year__ o none ☐

2-46) How many cakes or pastries did you eat?

N per day ___ o N per week __ o N per month_ o N per year__ o none ☐

Usually, what kind of sweets were they?

	Very often	often	sometimes	rarely	Never
cake stuffed with chocolate or creams					
stuffed cake (daisy, tart)					
pudding, tiramisu					
cookies					
brioche					
pastries (cream puff, cannoli)					

2-47) How many portions of corn-flakes, muesli, oatmeal did you eat?

N per day ___ o N per week __ o N per mo4nth_ o N per yea4r__ o none ☐

2-48) How many portions of chocolate (all kinds, including spreadable creams) of about 10 grams did you eat?

N per day ___ o N per week __ o N per month_ o N per year__ o none ☐

2-49) How many candies or spoonful of jam or honey did you eat?

N per day ___ o N per week __ o N per month_ o N per year__ o none ☐

2-50) How many ice creams did you eat (all kinds)?

N per day ___ o N per week __ o N per month_ o N per year__ o none ☐

2-51) Is there some food or some dish did you eat at least once a month and that was not included in this questionnaire?

☐ Yes ☐ NO

↓

If YES, please briefly describe:

____

____

____

____

2-52) Did you change your eating habits significantly in the last year?

☐ **Yes** ☐ **NO**

↓

If YES, for which reason?

☐ **to lose weight**

☐ **health reasons** *(please, specify)*______________________________

☐ **other** (please, specify) ______________________________

Section 3 – SMOKING HABITS

3-1) Do you currently smoke?

☐ **YES** ☐ **OCCASIONALLYE** **NO**☐

If YES:

How many cigarettes (cigars, pipe) do you smoke in a day? ______________

How old were you when you started smoking?____________________________

If OCCASIONALLY:

How many cigarettes (cigars, pipe) do you smoke in a week? _______or in a month?_________

How old were you when you started smoking?____________________________

If NO:

☐ I never smoked.

☐ I smoked in the past, now I stopped

How many cigarettes (cigar, pipe) did you smoke every day?__________

At what age did you start smoking?____________________________

At what age did you stop smoking?____________________________

Section 4 – GENERAL HEALTH

4-1) Did a medical doctor ever diagnosed one or more of the following conditions or diseases? (please answer any questions)

				Pharmacological therapy	
- High blood pressure	☐ YES	☐ NO	at what age? _______	☐ YES	☐ NO
- High cholesterol	☐ YES	☐ NO	at what age?_______	☐ YES	☐ NO
- High triglycerides	☐ YES	☐ NO	at what age?_______	☐ YES	☐ NO
- Diabetes	☐ YES	☐ NO	at what age?_______	☐ YES	☐ NO
- Malignant cancer	☐ YES	☐ NO	at what age?_______	☐ YES	☐ NO

If yes, where? ____

4-2) In addition to any previous diseases, should you regularly use drugs prescribed by a medical doctor? (excluding contraceptives)

☐ **YES** ☐ **NO**

If YES, for which disease? ____

____

4-3) Did you use oral contraceptives?

☐ **YES** ☐ **NO**

If YES, at what age did you start using them?_______________________________________

How old were you when did you stop using them? ______________________________
(if you already stopped)

Summing up all time periods, for how long did you take them?

☐ less than 1 month ☐ less then 1 year ☐ 2 years ☐ 3 years

☐ 4 years ☐ 5 years ☐ 6 years ☐ 7 years

☐ 8 years ☐ 9 years ☐ 10 years or more

4-4) Are you currently in menopause? (absence of regular period for at least 6 months)

☐ YES ☐ NO

If YES, at what age did you have your last menstrual period?___________

4-5) If YES, did you take or are you currently taking hormone replacement therapies to relieve menopause disorders?

☐ YES ☐ NO

If YES, at what age did you start using them? ____________________________

Summing up all time periods, for how long did you take them?

☐ less than 1 month ☐ less then 1 year ☐ 2 years ☐ 3 years

☐ 4 years ☐ 5 years ☐ 6 years ☐ 7 years

☐ 8 years ☐ 9 years ☐ 10 years or more

4-6) We also would like to ask you now some more detailed information on your reproductive history.

Did you have pregnancies?
☐ YES ☐ NO

If YES,

How many children did you have?__________

How many live-born children did you have?___________

Did you have spontaneous abortions or voluntary interruptions?
YES ☐ NO ☐

Did you breastfeed?
YES ☐ NO ☐

Please indicate the number of months of breastfeeding for each child:

1st child ____ 2nd child ____ 3rd child ____ 4th child ____

Section 5 – PSYCHOLOGICAL DISTRESS

What do you generally do when you experience adverse events or stressful situations? Please answer by marking an X on the correspondent answer.

Legend:
1 = I haven't been doing this at all.
2 = I've been doing this a little bit
3 = I've been doing this a medium amount
4 = I've been doing this a lot.

**5-1)**	I've been turning to work or other activities to take my mind off things.	1	2	3	4
**5-2)**	I've been concentrating my efforts on doing something about the situation I'm in.	1	2	3	4
**5-3)**	I've been saying to myself "this isn't real.".	1	2	3	4
**5-4)**	I've been using alcohol or other drugs to make myself feel better.	1	2	3	4
**5-5)**	I've been getting emotional support from others.	1	2	3	4
**5-6)**	I've been giving up trying to deal with it.	1	2	3	4
**5-7)**	I've been taking action to try to make the situation better.	1	2	3	4
**5-8)**	I've been refusing to believe that it has happened.	1	2	3	4
**5-9)**	I've been saying things to let my unpleasant feelings escape.	1	2	3	4
**5-10)**	I've been getting help and advice from other people.	1	2	3	4
**5-11)**	I've been using alcohol or other drugs to help me get through it.	1	2	3	4
**5-12)**	I've been trying to see it in a different light, to make it seem more positive.	1	2	3	4
**5-13)**	I've been criticizing myself.	1	2	3	4
**5-14)**	I've been trying to come up with a strategy about what to do.	1	2	3	4
**5-15)**	I've been getting comfort and understanding from someone.	1	2	3	4
**5-16)**	I've been giving up the attempt to cope	1	2	3	4
**5-17)**	I've been looking for something good in what is happening.	1	2	3	4
**5-18)**	I've been making jokes about it.	1	2	3	4
**5-19)**	I've been doing something to think about it less, such as going to movies, watching TV, reading, daydreaming, sleeping, or shopping	1	2	3	4
**5-20)**	I've been accepting the reality of the fact that it has happened	1	2	3	4
**5-21)**	I've been expressing my negative feelings	1	2	3	4
**5-22)**	I've been trying to find comfort in my religion or spiritual beliefs.	1	2	3	4
**5-23)**	I've been trying to get advice or help from other people about what to do.	1	2	3	4
**5-24)**	I've been learning to live with it.	1	2	3	4
**5-25)**	I've been thinking hard about what steps to take.	1	2	3	4
**5-26)**	I've been blaming myself for things that happened.	1	2	3	4
**5-27)**	I've been praying or meditating.	1	2	3	4
**5-28)**	I've been making fun of the situation	1	2	3	4

5-29) Do you think you (or persons around you) had traumatic experiences (e.g. grief, separation/divorce, work problems, health problems or people around you, etc.)?

☐ YES ☐ NO

If YES,

In which year did you have the last traumatic experience? ____________

In which year did you have the penultimate traumatic experience? ____________

Please, check having answering all questions!

Thanks for your collaboration!!!

The photo of foods were available by courtesy of Me.Te.Da. Srl.

Section 1 (physical activity) and section 2 (dietary habits) were modified from the EPIC (The European Prospective Investigation into Cancer and Nutrition) study questionnaires (http://epic.iarc.fr/).

Section 5 (psychological distresses) was taken from the Brief COPE questionnaire (http://www.psy.miami.edu/faculty/ccarver/sclBrCOPE.html).
